# Deep-rooted pigeon pea promotes the water relations and survival of shallow-rooted finger millet during drought—Despite strong competitive interactions at ambient water availability

**DOI:** 10.1371/journal.pone.0228993

**Published:** 2020-02-13

**Authors:** Devesh Singh, Natarajan Mathimaran, Thomas Boller, Ansgar Kahmen

**Affiliations:** Department of Environmental Science–Botany, University of Basel, Basel, Switzerland; Consiglio per la Ricerca e la Sperimentazione in Agricoltura, ITALY

## Abstract

Bioirrigation has been defined as the transfer of hydraulically lifted water by a deep-rooted plant to a neighbouring shallow-rooted plant which cannot access deep soil moisture. In this study, we tested if facilitative effects of bioirrigation or the competition for water dominate the interaction of two intercropped plants—deep-rooted pigeon pea (PP) and shallow-rooted finger millet (FM) before and during a drought. Additionally, we tested how the presence of a common mycorrhizal network (CMN) affects the balance between facilitative (i.e. bioirrigation) and competitive interactions between two intercropping species. Our results show that PP can indeed promote the water relations of FM during a drought event. Specifically, stomatal conductance in FM controls dropped to low values of 27.1 to 33.6 mmol m^-2^s^-1^, while FM in intercropping treatments were able to maintain its stomatal conductance at 60 mmol m^-2^s^-1^. In addition, the presence of PP reduced the drought-induced foliar damage and mortality of FM. The observed facilitative effects of PP on FM were partially enhanced by the presence of a CMN. In contrast to the facilitative effects under drought, PP exerted strong competitive effects on FM before the onset of drought. This hindered growth and biomass production of FM when intercropped with PP, an effect that was even enhanced in the presence of a CMN. The results from our study thus indicate that in intercropping, deep-rooted plants may act as "bioirrigators" for shallow-rooted crops and that a CMN can promote these facilitative effects. However, the interspecific competition between the intercropped plants under conditions of abundant moisture supply can be strong and are enhanced by the presence of a CMN. In more general terms, our study shows that the extent by which the antagonistic effects of facilitation and competition are expressed in an intercropping system strongly depends on the availability of resources, which in the case of the present study was water and the presence of biotic interactions (i.e. the presence of a CMN).

## Introduction

Deep-rooted plants can re-charge the topsoil layer through hydraulic lift (HL). HL is a process where water is transferred from deep moist soil layers to dry top soil layers through the roots of a plant as a consequence of a soil water potential gradient [1–4]. Deep-rooted plants performing HL could be used as a tool to recharge the topsoil layer in agricultural fields and possibly also to facilitate the transfer of the hydraulically lifted water to neighbouring shallow-rooted crops through “bioirrigation” [2,5]. Thus, bioirrigation could provide a simple and effective way to improve the water relations of shallow-rooted crops during drought in water-limited areas.

An early and strong evidence of bioirrigation in cropping systems comes from work of Corak et al. [6], where tritium-labelled water lifted by alfalfa plants was transferred to neighbouring maize plants, resulting in prolonged survival of maize plants during drought in an experimental greenhouse study. Sekiya and Yano [7] conducted a field study to demonstrate that maize plants grown near pigeon pea were able to utilize water that was hydraulically lifted by pigeon pea. In a recent study, Bogie et al. [8] showed that during experimentally-imposed drought, shallow-rooted pearl millet (*Pennisetum glaucum*) was able to take up water hydraulically lifted by a deep-rooted shrub (*Guiera senegalensis*), and as a consequence millet biomass production when intercropped with shrubs was over 900% greater than millet biomass in monoculture. Similarly, in an agroforestry set up, Hirota et al. [9] showed that upland rice (*Oryza sativa*) plants grown in split-root systems with a markhamia tree (*Markhamia lutea*) were viable and green during a drought period, while rice plants alone could not survive. These studies indicate the potential of bioirrigation to provide water to shallow-rooted crops when these are intercropped with deep-rooted plants.

While facilitative effects of bioirrigation might support the water relations and survival of shallow-rooted crops, two plant species placed in close vicinity in intercropping systems can also compete with each other for resources such as light, nutrients and particularly soil moisture with impacts on growth and yield of the individual plants [10–12]. Ludwig et al. [13] reported that grasses interspersed with *Acacia tortilis* were able to take up water hydraulically lifted by *Acacia*. However, the biomass production of grasses was higher in trenched plots (grass-tree root systems separated) than in grasses that had their roots interspersed with *Acacia*. Reduced growth was thus the result of below-ground competition for water that overwhelmed the facilitative effects of bioirrigation during drought periods. Similarly, Zegada-Lizarau et al. [14] showed that pearl millet (*Pennisetum glaucum*) in intercropping with cow pea (*Vigna unguiculate*) had lower leaf water potential (under drought) and biomass than in monoculture due to competition. To make bioirrigation effective for the promotion of yield in intercropping system, it is thus important to assess how facilitative and competitive effects between the two co-occurring plants interact in the overall determination of yield [15].

A further important limitation for the facilitative potential of bioirrigation is the distance between the rhizosphere of two plants. Efflux of HL water from one plant is usually tightly held up in the rhizosphere of the same plant [16] and an effective transfer of water between two plants is hindered by the distance of their rhizospheres. Arbuscular mycorrhizal fungi (AMF) could provide a pathway for the transfer of water between two plants via a common mycorrhizal network (CMN), thereby facilitating bioirrigation [17]. In a recent study, Saharan et al. [18] showed that the presence of a CMN between pigeon pea (PP) and finger millet (FM) alleviates the negative effect of drought on finger millet, suggesting that a CMN can connect the rhizospheres of two plants and thereby facilitate bioirrigation. Furthermore, Egerton-Warburton et al. [19] showed the AMF facilitated transfer of water (released as HL efflux) from coastal live oak seedlings to water-stressed oak seedlings. Similarly, in a recent study, we [20] report that the presence of a CMN facilitated the transfer of HL water from deep-rooted PP to shallow-rooted FM, and that bioirrigated FM was able to maintain its stomatal conductance under drought. These studies present accumulating evidence that AMF could indeed promote bioirrigation.

Importantly, however, the presence of a CMN can significantly affect the competitive interaction between different plant species [21,22]. Weremijewicz and Jonas [23] reported that, in the absence of root system overlap, CMN promotes asymmetric below ground competition and that a CMN may benefit large individuals at the expense of small plants. Previous studies testing the effects of CMN on bioirrigation have largely focussed on identifying facilitative aspects of CMN-mediated plant-plant interaction. To determine the balance between positive (facilitative) and negative (competitive) effects in an intercropping system, it is however, not only necessary to identify bioirrigation as a process but also to quantify its effect on yield of the involved species. The balance between positive and negative effects in CMN-facilitated intercropping systems has previously not been quantified, possibly because most previous studies have tested the effects of CMN-facilitated bioirrigation utilizing rather smaller pot sizes [18,20,24,25]. However, in small pots reduced plant growth [26] cannot fully inform on the quantitative facilitative and competitive interactions between two plants as they would occur in the field. In addition, drought periods simulated in small pots are often not realistic because of the rapid pre-emption of the soil water reservoir in small pots preventing the possibility to maintain moderate but realistic drought situations for extended period of time.

In the current study, we tested how the presence of a CMN affects the balance between facilitative (i.e. bioirrigation) and competitive interactions between two intercropping species. We used an established intercropping system of deep-rooted PP and shallow-rooted FM to quantify CMN-affected competitive and facilitative interactions with respect to water relations and bioirrigation. We aimed to conduct this study under controlled conditions and designed an intercropping system in a cylindrical large pot of 50 L to address following specific research questions: (i) Does the presence of PP as bioirrigator result in interspecific competition for water with FM before and during drought conditions? (ii) How are the competitive interactions between PP and FM influenced by a CMN network? (iii) Does PP support the water relations and survival of neighbouring shallow-rooted FM through bioirrigation during drought? (iv) Can a CMN facilitate of bioirrigation? (v) Does the balance between competitive and facilitative effects lead to an increase or reduction of yield in a CMN-facilitated intercropping system of PP and FM?

## Material and methods

### Experiment set up

To test the potential of bioirrigation in intercropping systems of deep-rooted PP and shallow-rooted FM under drought, a pot experiment was established inside a greenhouse under controlled conditions (14 hrs of day light with PPFD 350 to 400 μMol/S at 26±5°C and 10 hrs of dark (night) duration at 20±5°C and 60±10% relative humidity) at the University of Basel, Switzerland.

**Fig 1:**
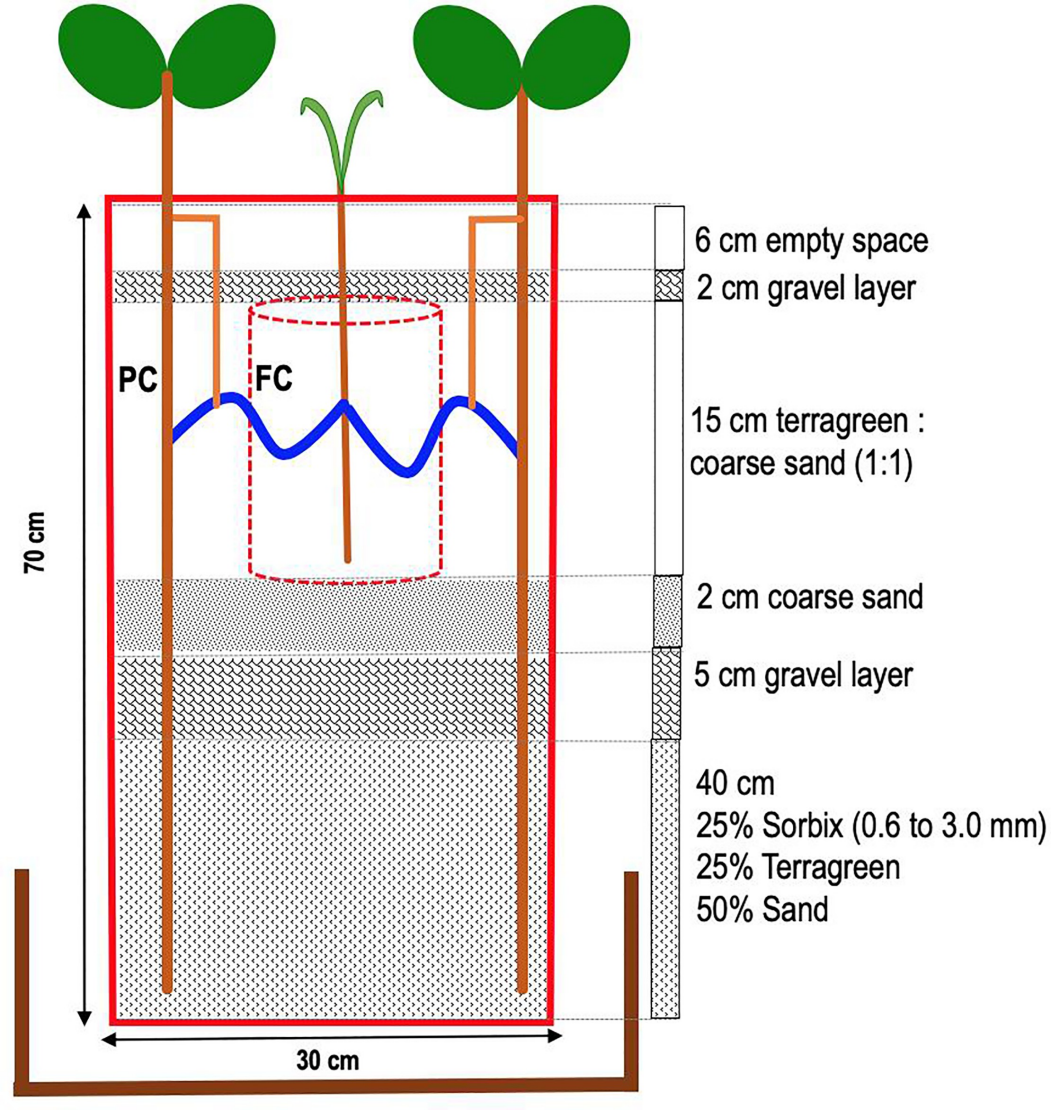
Pot set up. A main pot with a dimension of 70 x 30 cm (h x d) was filled with different layers of soil, sand and gravel. A finger millet compartment (FC) was made with nylon mesh (21 μm pore size) to restrict the growth of plant roots but allow mycorrhizal hyphae to pass through and connect PP and FM roots. The FC contained two FM plants per pot. The pigeon pea compartment (PC) contained two PP plants per pot. The main pot was placed into a wider pot of 31 x 45 cm (h x d) to water the main pot from the bottom during the drought treatment (see also Fig 2).

PP and FM plants were grown in PVC pots of 70 cm height and 30 cm diameter. The pot design was inspired by Saharan et al. [18], but the pots had a much larger height and volume. In brief, each pot was filled with layers of different substrate materials (sand, gravels and terragreen) as shown in Fig 1. The bottom layer of each pot (40 cm) contained a mix (1:1:2) of sorbix (0.6–3.0 mm), terragreen and fine sand (0.1–0.4 mm) which has the capacity to hold water in order to serve as a source of water for the deep-rooted PP. Above the bottom layer a gravel (2–4 mm) layer of 5 cm was installed with the purpose to prevent the capillary rise of water from the bottom layer to the top layer of the pot. Above the gravel layer, there was a 2 cm layer of medium fine sand (1–2 mm). The top layer of 15 cm was filled with mix (1:1) of terragreen and fine sand. The top layer was divided into two compartments: A central compartment for FM, which was 12 cm wide and 15 cm deep. The compartment was made of a nylon mesh (21 μm pore diameter, Anliker AG, Basel, Switzerland) to restrict roots of FM to grow outside the compartment. In addition, pots contained an outer compartment for PP, where roots were allowed to reach the bottom layer of the pot. All sand (purchased from Quratz dÀlsac LA France) and terragreen (Maagtechnic AG Dübendorf, Switzerland) material used in this experiment was sterilized by heating at 80°C for 12 hours prior to the experiment.

**Fig 2:**
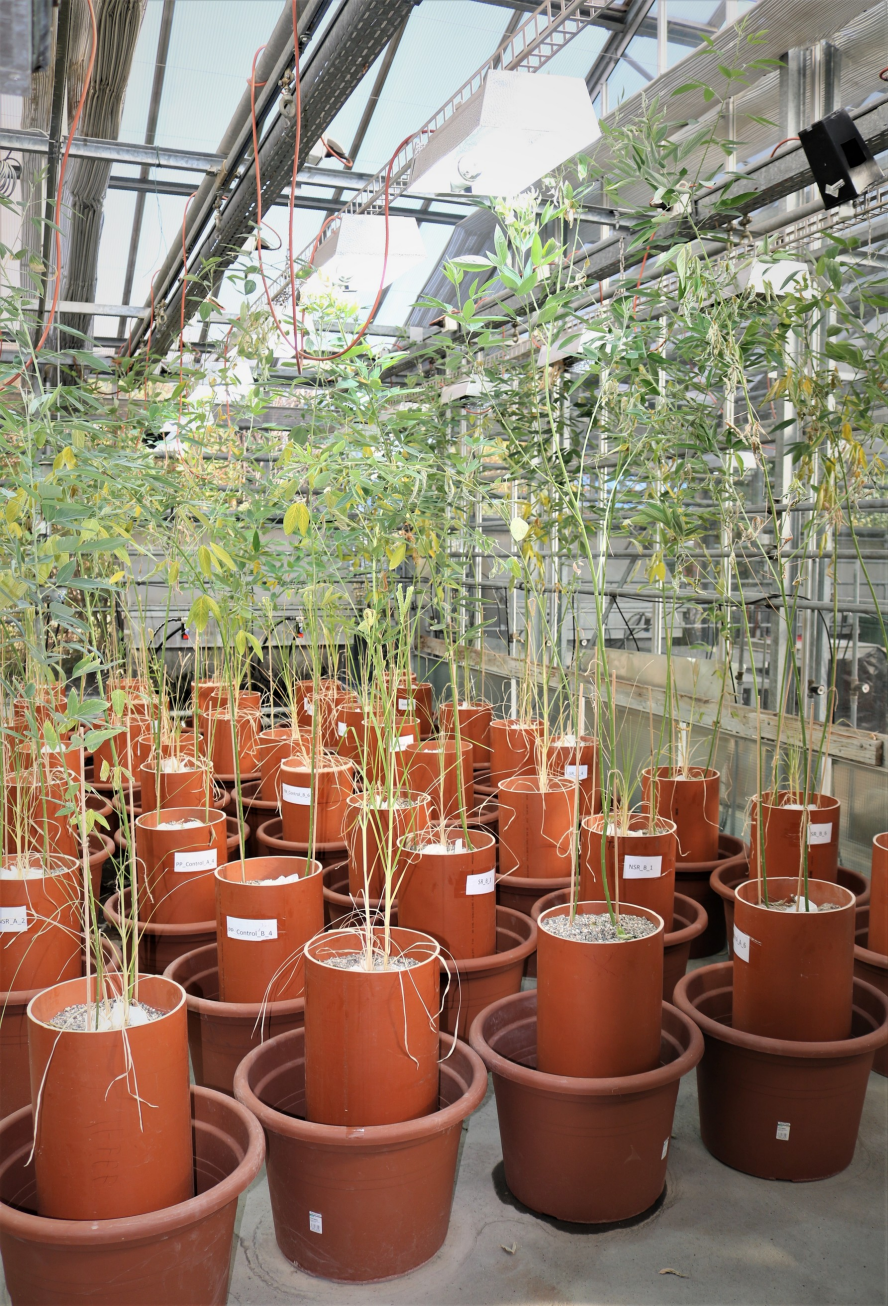
Experimental set up. A two-pot set up was used to grow the plants, where the outer pot was used as reservoir to water the main pot from the bottom layer during the drought treatment. The picture was taken towards the end of drought period.

Each 70 cm x 30 cm (h x d) main pot was placed into a wider pot of 31 x 45 cm (h x d). This outer pot had a valve at the bottom. This allowed us to fill and drain the outer pot with water in order to water the main pot from bottom during the drought treatment (see Fig 2).

Plants were fertilized with 50 ml of modified Hoagland solution (with P content 75% reduced) every third week until beginning of the drought period. The Hoagland solution was reduced in P because a low P content is required for AMF to be actively involved in nutrient mobilisation and colonization of plant roots [27].

**Fig 3:**
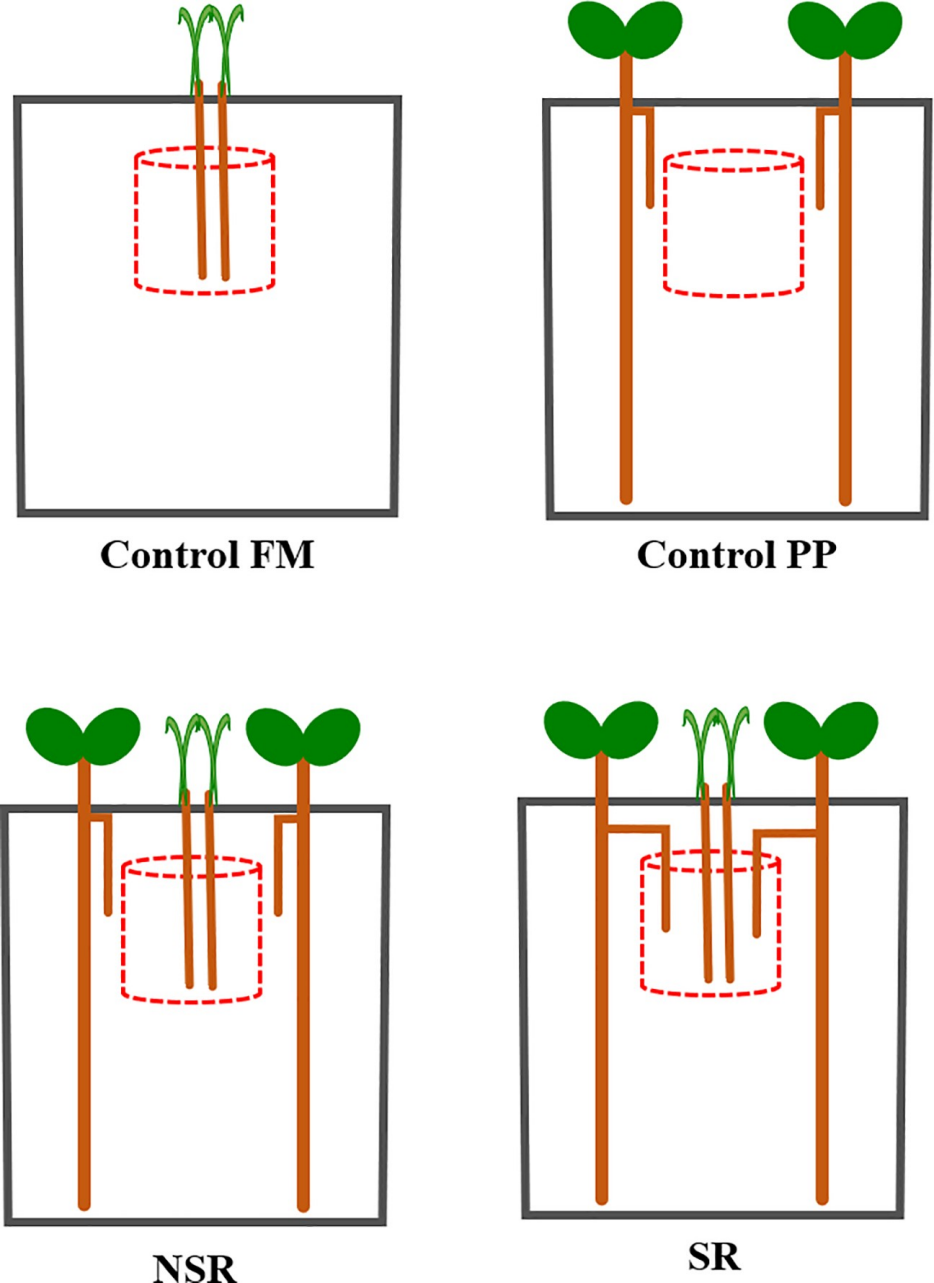
Experiment design with different treatments. The study included eight different treatments: FM monoculture without and with CMN, PP monoculture without and with CMN, non-split-root (NSR) treatment without and with CMN, and split-root (SR) treatment without and with CMN. In the split-root (SR) treatment, lateral roots of PP plant were inserted into the FM compartment. Monoculture treatments had two PP plants in the PP control and two FM plants in the FM control. Intercropping treatments had two PP plants and two FM plants. Thus, the intercropping system we tested followed an “addition design”.

We installed eight different treatments with five replicates per treatment in our experiment: monoculture of FM and PP with and without CMN as control, non-split-root (NSR) treatment and split-root (SR) treatment with and without CMN (Fig 3). In the SR treatment, lateral roots of PP were inserted into the FM compartment to allow intermingling of roots and to facilitate the direct transfer of water efflux from PP roots. NSR and SR treatments were established to study if root interactions (i.e. intermingling of two roots) could enhance the transfer of hydraulically lifted water (HLW) from PP to FM.

### Plant material

The deep rooting plant used in this study was PP (*Cajanus cajan* cv. BRG2) and the shallow-rooted plant was FM (*Elusine coracana* cv. GPU28). Seeds were sterilized by shaking seeds for 2 minutes in a 1% sodium hypochlorite (NaOCl) solution [28]. PP seeds were pre-germinated into a 50 cm long tube (5 cm diameter) that was filled with four different layers: a bottom layer of 10 cm with a mix (1:1) of fine sand (0.1–0.4) and terragreen. Above this was a gravel (2–4 mm) layer of 15 cm and a layer of medium fine sand (1–2 mm). The top layer of 15 cm was filled with a mix (1:1) of fine sand and terragreen. The pre-germination of seedlings was done to ensure PP roots to reach the bottom layer of the main pots. This was necessary as PP seeds directly germinating in the main pots did not grow to the bottom of the pot, possibly due to the high physical resistance of the gravel layer at 17 cm depth. Pre-grown PP were then carefully taken out of the tube and transplanted into the main pots after 45 days, when roots were about 50 cm long. The day when PP seeds were sown for germination was counted as "day of experiment one" (DOE 1). In order to keep the age difference between PP and FM not more than 30 days, FM seeds were germinated, at DOE 30, in a tray filled with a mix (1:1) of fine sand (0.1–0.4) and terragreen. The age difference of 30 days between PP and FM plant is a common intercropping practice for these species where farmers are recommended to use four to five week old PP seedlings to transplant and sowing of FM seeds immediately for higher yields [29]. Both 15 days FM and 45 days PP seedlings were transferred into the experimental main pots on DOE 45. Monoculture treatments had two plants of FM or PP, while all intercropping treatments had two plants of FM in the central FM compartment and two plants of PP in the outer compartment.

### Bioinoculants

To establish a CMN between PP and FM, AMF strains of *Rhizophagus fasciculatus*, formerly called *Glomus fasciculatum* (63 spores per 10 g substrate), and *Ambispora leptoticha*, formerly called *Glomus leptotichum* (67 spores per 10 g), cultured in Rhodes grass roots, were used as inoculants. To ensure nodulation of PP, we also used the Rhizobia strain *Bradyrhizobium* sp. (DSMZ-5969, Leibniz Institute DSMZ-German Collection of Microorganism and Cell Cultures, Germany). In addition, two PGPR (Plant Growth Promoting Rhizobacteria) strains (*Pseudomonas fluorescens* strains R62 and R81) were used [30]. PGPRs are known to have beneficial effects on plant growth, especially in the development of fine root growth [31]. Treatments without AMF did not receive *Bradyrhizobium sp*. and two PGPR strains. However, a soil wash from a natural field site was added to provide a natural microbiome and ensure nodulation in PP.

AMF treatments were inoculated with 5 g AMF culture per plant, and 2 ml of bacterial inoculum containing 1x10^6^ cfu/ml were added. In all CMN treatments, the *Rhizophagus fasciculatus* culture was placed in the FM compartment, while the *Ambispora leptoticha* cultured was placed in the PP compartment. 2 ml of the bacterial inoculum was added into FM and PP compartment in all CMN treatments. The AMF strains *Rhizophagus fasciculatus* and *Ambispora leptotichua* used in this study have been found to have more symbiotic efficiency for FM and PP, respectively, than other AMF strains [32,33]. Treatments without CMN were given AMF wash and cell free broth. In order to provide a natural microbiome in all treatments soil wash (soil collected from field site used for pigeon pea and finger millet intercropping at University of Agricultural Sciences, Bengaluru, India) was added into all pots. Soil and AMF wash was prepared separately by dissolving 50 g of each component in 1000 ml of tap water and the solution was filtered three times using Whatmann No. 1 filter paper.

### Watering and drought treatment

During the pre-drought period pots were watered once a week with 3 litres of tap water from the top to saturate the entire pot. Watering a volume of 3 litres was decided by measuring the amount of water required to saturate the entire pot. Pots had drainage holes at the bottom that allowed drainage of excess water. Watering from the top was done until PP roots established a good connection to the bottom layer of the pot. This was checked by destructively inspecting additional pots that were established for this purpose. In order to start the drought period, watering was gradually reduced to 1.5 litre, 1.0 litre and 500 ml on DOE 147, 154 and 161, respectively. The full drought period then started from DOE 168. During the drought period, pots were watered by submersing only the bottom part of the main pots by up to 25 cm in tap water (Figs 1 and 2) for two minutes, once a week. To submerse the main pot up to 25 cm in water, the outer pot was filled with water up to a height of 25 cm. Drainage holes at the bottom of the outer pot enabled complete drainage of water after 2 min to keep the pot set up completely dry. The drought period continued till FM in all control treatments had died (DOE 245).

### AMF root colonization

To analyse the root colonization by AMF, aliquots of fresh root material from PP and FM were harvested at the end of the experiment. Small pieces of roots were cut from the entire root system and mixed together to get a homogenized sample. Samples were stored in 50% ethanol. For the assessment of root colonization by AMF, root segments were cleared in KOH (10%, w/v; at 4°C, 1 week) and stained with trypan blue (0.05% w/v, at room temperature, 6 h). Root segments were de-stained, and 25 randomly selected segments were observed for the presence or absence of AMF functional structures (hyphae, vesicles and arbuscules). The percent root colonization was then calculated according to Brundrett [34] by examining 100 intersections on 25 randomly selected root fragments for each root sample.

### Physiological and growth parameters

To monitor the water relations of FM, stomatal conductance (gs) was measured at mid-day between 12:30 to 14:30 hours, using an SC-1 leaf Porometer (Decagon Devices, USA). Measurements were done 48 hours after watering from DOE 147 to 245 of the experiment. Central leaves of FM were selected for measurements, and two leaves per plant were measured on the upper surface. Soil moisture in the top layer of the pot was also measured on the same day using a ML3 theta probe (Delta-T Devices, Cambridge, UK) every week from DOE 182 onwards (drought started on DOE 168) till DOE 245. Soil moisture probes were placed into the topsoil layer (up to 6 cm to completely immerse the needles) in the PP compartment close to the FM compartment to avoid damage of the root network inside FM compartment. To observe growth during the experimentally induced drought period, plant height of FM was measured every week from DOE 161 onward till end of the experiment i.e. DOE 245.

### Assessment of total foliar damage and biomass at harvest

Foliar damage of FM plants was measured at the end of experiment on DOE 245. Foliar damage was assessed by counting the number of dead leaves on a plant. A leaf was defined as dead when less than one third of its length was green/yellowish green, and the remainder was desiccated. Percent foliar damage was calculated as number of dead leaves/total number of leaves*100. A plant was defined as dead when all leaves were desiccated (as defined above) and no signal for stomatal conductance was recorded. Dead plants were not removed from the pots until the end of the experiment.

At the end of the experiment, shoot and root parts of each plant were separately harvested. First the FM compartment was removed and shoot and root parts were separated. After this the PP plants were harvested. The roots were washed with tap water to remove sand particles. For determining dry biomass, shoot and root samples were kept in paper bags at 80°C in a hot air oven (model UF260, Memmert GmbH + Co. KG, Germany) for 48 hours.

#### Land equivalent ratio (LER)

The facilitative and competitive interactions between PP and FM in response to the different treatments were calculated using the land equivalent ration (LER). The LER was calculated as [35]:

$$LER=LER_{FM}+LER_{PP}$$

$$LER_{FM}=(\frac{Y_{FM,PP}}{Y_{FM}}),\;LER_{PP}=(\frac{Y_{PP,FM}}{Y_{PP}})$$

Where Y_FM_ and Y_PP_ are yield of PP and FM in its monoculture, Y_FM,PP_ is yield of finger millet in intercropping, and Y_PP,FM_ is yield of pigeon pea in intercropping. The baseline for LER is one. If the LER is greater than one intercropping favours growth and yield of plants, and when it is lower than one intercropping negatively affect the growth and yield of plants.

### Statistical analysis

Data throughout the manuscript are expressed as mean ± one standard error of the mean (SEM). Treatment effects on total biomass of FM and PP were tested separately for each species by one-way ANOVAs using tukey’s test for post hoc multiple treatment comparison. To compare the general effects of monocropping vs intercropping and CMN on foliar damage and total FM and PP biomass, nested treatments were categorized as either monocropping (control treatments: FM-, FM+, PP- and PP+) or intercropping (NSR-, NSR+, SR- and SR+) with and without CMN and were tested using two-way ANOVAs. To compare the effects of root interaction vs. no root interaction and CMN on foliar damage and total FM or PP biomass within intercropping, treatments were categorized as either root interaction (SR- and SR+) or no root interaction (NSR- and NSR+) with and without CMN, and were tested using two-way ANOVAs. The criterion for significance was p<0.05. GraphPad Prism software (version 7.0 for Mac OS X, GraphPad Software, La Jolla California USA) was used to perform statistical analysis.

## Results

### AMF colonization

**Fig 4:**
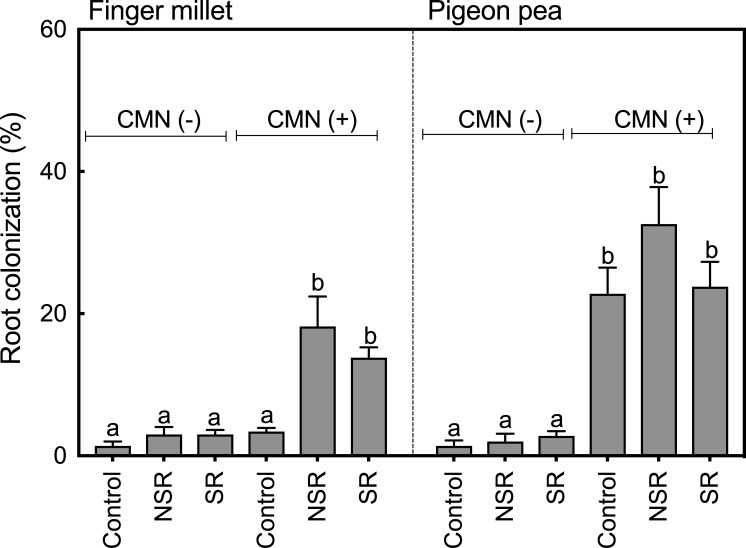
Colonization of FM and PP roots by AM fungi. Bars represent the average of five replicates with one standard error of the mean. Tukey`s test was used for multiple comparison (PP and FM separately) and values with same letters are not significantly different at p>0.05. Treatments with AMF and PGPR additions are represented with CMN (+), and without AMF and PGPR additions as CMN (-). Control represents the monoculture treatments, while NSR and SR represent non-split root and split root treatments of the intercropping treatments, respectively.

Root colonization data show that plants in the treatments without AMF and PGPRs (CMN (-)) had very low root colonization rates that ranged from 1.4% to 3.0% in FM and 1.4% to 2.8% in PP (Fig 4). Also, FM roots in the monoculture CMN (+) treatment with added AMF and PGPRs had similarly low colonization rates (Fig 4). In the CMN (+) intercropping treatments, FM had significantly more roots colonized by AMF than in monoculture, with colonization rates ranging from 13.2% to 18.2% in SR and NSR treatments, respectively. PP plants in the CMN (+) treatments had an about ten-fold higher colonization compared to plants in the CMN (-) treatment, both in monoculture and in intercropping treatments.

### Water relations of finger millet during drought

**Fig 5:**
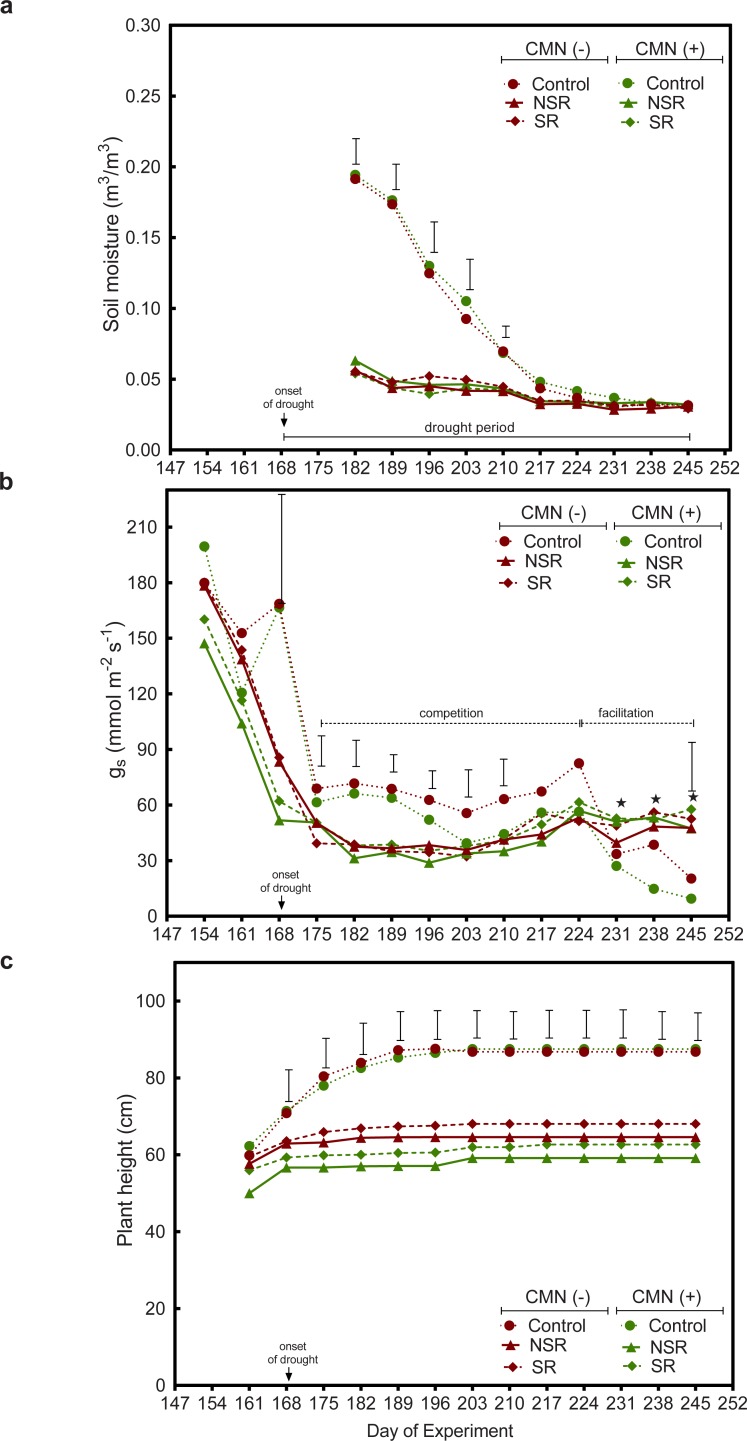
Soil moisture of the topsoil layer of all treatments (Fig 5A), stomatal conductance (g_s_, Fig 5B) and plant height of FM during drought (Fig 5C). Treatments with AMF and PGPR additions are represented as CMN (+), and without AMF and PGPR additions as CMN (-). Control represents monoculture, while NSR and SR represent non-split root and split root treatments of intercropping, respectively. Values shown in the graph are the average of five replicates and bars represent Tukey`s HSD_0.05_ value above data with significant differences among treatments. The drought period started at day 168 of the experiment. Star (↔) symbols in Fig 5B represent data points for control treatments where some replicate plants did not survive. Only living plants were used to measure stomatal conductance (see Table 3).

Two weeks after the onset of the drought treatment (DOE 182), soil moisture in the topsoil layer in all intercropping treatments had already declined to a value of ca. 0.06 m^3^/m^3^ (Fig 5A). This was independent of the presence of a CMN. In contrast, in the monoculture (control) treatments of FM with or without CMN, soil moisture was 0.19 m^3^/m^3^, which was significantly higher than in the intercropping treatments two weeks after the onset of drought. Soil moisture in the topsoil layers in the control treatments gradually decreased as the drought period prolonged, reaching 0.05 m^3^/m^3^ at DOE 217, which was similar to the values observed in the intercropping treatments.

The response of stomatal conductance (g_s_) during the drought in the different treatments can be separated into three phases (Fig 5B). Phase 1 (before and at the onset of drought DOE 154–168): all treatments had similar values for g_s_, ranging from 147.2 to 199.5 mmol m^-2^s^-1^ at DOE 154 before the drought started. With the onset of drought we observed that g_s_ declined in all treatments (DOE 175). This decline was more rapid in the intercropping treatments than in the monocropping controls and independent of the presence of a CMN. Phase 2 (progression of drought DOE 175–224): FM in the control and intercropping treatments maintained a low yet stable g_s_ for seven weeks. In general, g_s_ was higher in the monocropping treatments than gs in the intercropping treatments and this effect was mostly independent of CMN. Phase 3 (end of drought DOE 224–245): at DOE 231, g_s_ in the controls dropped to low values ranging from 27.1 to 33.6 mmol m^-2^s^-1^, while g_s_ in the intercropping treatments was maintained at values around 60 mmol m^-2^s^-1^ (Fig 5B). As such, FM in intercropping treatments maintained a low but consistent gas-exchange until the end of the drought treatment, while g_s_ of FM in monoculture treatments (with and without CMN) dropped to very low values ranging from 9.4 to 20.3 mmol m^-2^s^-1^ at DOE 245. The presence of a CMN did not show any effect on stomatal conductance of FM in intercropping treatments (NSR and SR) or controls during phase 3.

### FM growth

Before the onset of drought, at DOE 161, plant height of FM was similar in all treatments (Fig 5C). In the first weeks after the onset of drought (DOE 168), plant height increased more in the control (monoculture) treatments than in the intercropping treatments. This trend continued until 4 weeks after the onset of drought. From DOE 196 onwards, there was no increase in plant height in monoculture. While, FM in all intercropping treatments did not grow in height one week after the onset of drought. FM plants in monoculture with or without CMN had significantly higher plant height than FM in the intercropping treatments. The presence of a CMN did not have any positive effect on plant height of FM in the controls but FM plants in the intercropping treatments were slightly higher, when a CMN was absent.

### Foliar damage of FM in response to drought

**Fig 6:**
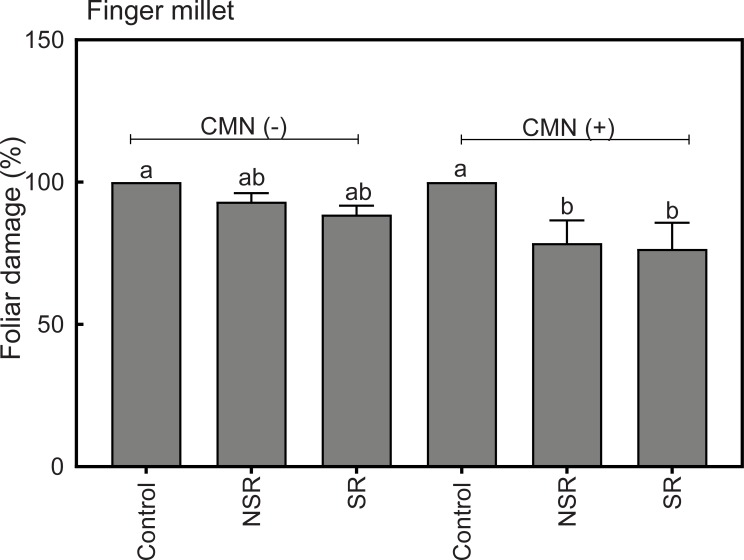
Percent foliar damage of FM at day 245 of the experiment. Treatments without or with AMF and PGPR application are shown as CMN (-) and CMN (+), respectively. Control represents monoculture, while NSR and SR represent non-split root and split root treatments of intercropping, respectively. Bars show average values of five replicates with one standard error of mean. Tukey`s test was used for multiple comparison. Values with the same letters indicate no significantly different values at p>0.05. For a general analysis of treatment effects see Tables 1 and 2.

FM in control treatments with and without CMN showed 100% foliar damage at the end of the experiment (DOE 245). FM in intercropping treatments (NSR and SR) showed lower damage rates of 78.5% and 76.5% with CMN, and 93.1% and 78.5% without CMN, respectively, than FM in monoculture (Fig 6). Damage rates in intercropping treatments differed significantly due to the presence of a CMN (Table 1), while root interaction (i.e. SR and NSR treatments) within intercropping treatment did not have a significant effect on the foliar damage rates (Table 2). FM plants in intercropping treatments had 100% survival rate (Table 3). In contrast, only 1 and 2 replicates of FM were alive in monocultures with and without CMN, respectively, at the end of drought period (DOE 245).

**Table 1:** Two-way ANOVA showing the effects of monocropping vs. intercropping and presence of a CMN on foliar damage in all treatments.

	SS	DF	F	P value
Monocrop vs. intercrop	1717	2	6.158	P = 0.0069
CMN (-) vs. CMN (+)	594.4	1	4.264	P = 0.0499
Interaction	304.9	2	1.093	P = 0.3512

**Table 2:** Two-way ANOVA showing the effect of root treatments (split roots (SR) vs. non-split roots (NSR)) and presence of a CMN on foliar damage in intercropping treatments.

	SS	DF	F	P value
SR vs. NSR	53.79	1	0.2572	P = 0.6190
CMN (-) vs. CMN (+)	891.6	1	4.264	P = 0.0555
Interaction	7.688	1	0.03676	P = 0.8504

**Table 3:** Number of surviving FM plants between days of experiment (DOE) 224 and 245 when all treatments experienced similar levels of drought. FM in all five replicates of intercropping treatments were alive throughout the experiment, while in FM control treatments started to die from DOE 231 to 245.

Treatment	CMN	DOE 224	DOE 231	DOE 238	DOE 245
Control	Yes	5	3	2	1
Control	No	5	3	3	2
NSR	Yes	5	5	5	5
NSR	No	5	5	5	5
SR	Yes	5	5	5	5
SR	No	5	5	5	5

### Biomass of FM and PP

**Fig 7:**
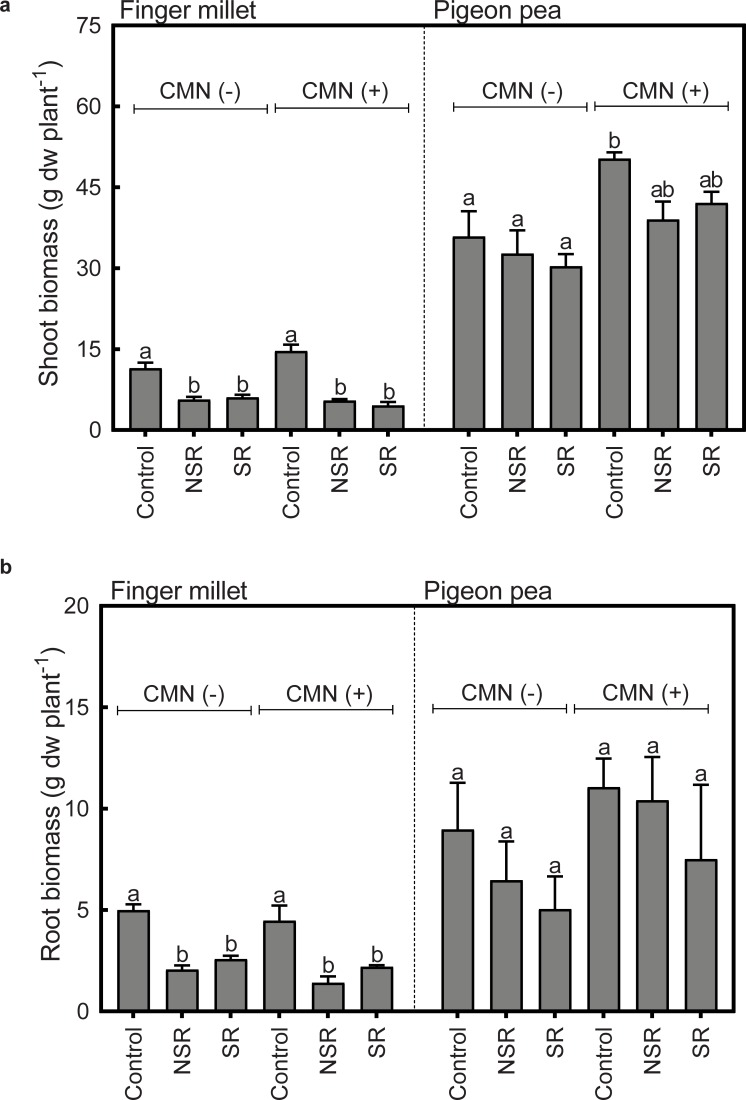
Shoot and root dry biomass of FM and PP in different treatments. Treatments without or with AMF and PGPR application are shown as CMN (-) and CMN (+), respectively. Control represents monoculture, while NSR and SR represent non-split root and split root treatments of intercropping, respectively. Bars represent average dry weight (dw) of five replicates with one standard error of the mean. Tukey`s test was used for multiple comparison (PP and FM separately) and values with same letters are not significantly different at p>0.05. For a general analysis of treatment effects see Tables 4 and 6.

In general, shoot and root biomass of FM plants was significantly lower in intercropping treatments as compared to monocropping treatments (Fig 7). Two-way ANOVAs showed that FM total shoot and root biomass was significantly reduced by intercropping and that the presence of a CMN did not have a significant overall effect on FM total biomass (Table 4). However, within intercropping treatments (NSR and SR) the presence of a CMN had a significant negative effect on FM total biomass, but root interaction treatments (i.e. connecting the lateral roots of FM and PP in the SR treatment) did not have a significant effect on FM total biomass (Table 5). In case of PP, a two-way ANOVA (Table 6) showed that PP total biomass was significantly reduced in the intercropping but the presence of a CMN had a general biomass enhancing effect. While, within intercropping treatments, root interaction treatments had no significant effect on PP total biomass (Table 7), but the presence of a CMN showed a significant biomass enhancing effect between SR and NSR treatments. with root interactions (SR) and no root interaction (NSR).

**Table 4:** Two-way ANOVA showing the effects of monocropping vs. intercropping with and without CMN on FM total biomass (shoot and root) in all treatments.

	SS	DF	F	P value
Monocrop vs. intercrop	708.6	2	71.14	P<0.0001
CMN (-) vs. CMN (+)	0.00147	1	0.00029	P = 0.9864
Interaction	28.61	2	2.872	P = 0.0762

**Table 5:** Two-way ANOVA showing the effects of root treatments (split roots (SR) vs. non-split roots (NSR)) with and without CMN on FM total biomass (shoot and root) in intercropping treatments only.

	SS	DF	F	P value
SR vs. NSR	0.8161	1	0.5067	P = 0.4868
CMN (-) vs. CMN (+)	9.275	1	5.759	P = 0.0289
Interaction	1.352	1	0.8394	P = 0.3732

**Table 6:** Two-way ANOVA table showing the effects of monocropping vs. intercropping with and without CMN on PP total biomass (shoot and root) in all treatments.

	SS	DF	F	P value
Monocrop vs. intercrop	642.3	2	3.968	P = 0.0324
CMN (-) vs. CMN (+)	1403	1	17.34	P = 0.0003
Interaction	49.92	2	0.3084	P = 0.7375

**Table 7:** Two-way ANOVA showing the effects of root treatments (split roots (SR) vs. non-split roots (NSR)) with and without CMN on PP total biomass (shoot and root) produced in intercropping treatments only.

	SS	DF	F	P value
SR vs. NSR	16.06	1	0.2111	P = 0.6521
CMN (-) vs. CMN (+)	750.6	1	9.869	P = 0.0063
Interaction	19.44	1	0.2557	P = 0.6200

### Land Equivalent Ratio

LER values indicate that all intercropping treatments showed a trend of total biomass gain (Table 8). However, only treatments without CMN showed significantly higher LER values than sole crop but no significant differences between LER values in treatments with and without CMN were observed. A two-way ANOVA (Table 9) shows that biomass gain in the intercropping treatments was not significantly affected by root treatments (i.e. in NSR and SR treatments) and the presence or absence of a CMN was marginally not significant (p = 0.0711).

**Table 8:** LER of different treatments. Total biomass (shoot + root) of FM and PP were used to calculate and compare the LER of each treatment. Only the treatments NSR and SR (without CMN) showed significantly (p<0.05) higher LER values than sole crops (i.e. control of PP and FM).

CMN inoculation	Treatment	LER (FM)	LER (PP)	LER (FM+PP)
CMN(-)	NSR	0.48±0.10	0.89±0.28	1.37±0.29*
	SR	0.53±0.10	0.83±0.23	1.36±0.26*
CMN (+)	NSR	0.38±0.11	0.81±0.11	1.19±0.14
	SR	0.36±0.08	0.81±0.10	1.17±0.13

**Table 9:** Two-way ANOVA showing the effects of root treatments (split roots (SR) vs. non-split roots (NSR)) and the presence or absence of a CMN on LER (total biomass), on partial LER of FM, PP, and total LER.

LER (FM)	SS	DF	F	P value
SR vs. NSR	0.002205	1	0.2243	0.6422
CMN (-) vs. CMN (+)	0.09385	1	9.544	0.0070
Interaction	0.006125	1	0.6229	0.4415
**LER (PP)**	**SS**	**DF**	**F**	**P value**
SR vs. NSR	0.004500	1	0.1141	0.7399
CMN (-) vs. CMN (+)	0.01352	1	0.3429	0.5663
Interaction	0.005120	1	0.1299	0.7233
**Total LER (FM+PP)**	**SS**	**DF**	**F**	**P value**
SR vs. NSR	0.0007200	1	0.01523	0.9033
CMN (-) vs. CMN (+)	0.1767	1	3.738	0.0711
Interaction	0.0000844	1	0.00162	0.9677

## Discussion

Our results indicate that in a PP-FM intercropping system, bioirrigation supports the water relations of FM during drought and helps FM to survive the drought period. However, our data also show that FM in intercropping treatments tends to face drought conditions earlier than in monoculture due to apparent competition with PP for water in the topsoil layer. This can explain the lower total biomass of FM in the intercropping treatments as compared to the monocrops despite bioirrigation during drought. The establishment of a CMN enhanced both, the facilitative effects of PP on FM during drought (as evident from reduced foliar damage) but also the competitive interactions between FM and PP before the drought, which became evident from reduced FM growth in the intercropping treatments that included a CMN.

### Colonization by AMF

In this study, a combination of AMF strains, rhizobia and PGPR were used to develop a CMN between rhizosphere of PP and FM. Previous studies [36,37] have reported that AMF colonization is more effective in legumes when they are nodulated by N_2_-fixing bacteria. PP and FM roots have been reported to show up to 65% and 75% colonization, respectively [18], while in our study, we observed AMF colonization of only 32.6% in PP and 18.2% in FM. Similar to this, Beggi et al. [38] has also reported low colonization rates in millets that varied from 12% to 30%. As root colonization is affected by the soil type and its nutrient content, the low colonization percentage in PP and FM roots in our set up could possibly be due to high soil P, since typically low colonization percentage have been reported under high soil P conditions [39,40]. In our experimental set up, the source of high soil P could be the terragreen substrate that contains up to 0.1% of P_2_O_5_ (technical data from manufacturer).

### Facilitation of water supply to FM during drought via bioirrigation

Our results suggest the facilitation of water to FM by PP during prolonged drought periods, so that FM plants in all intercropping treatments maintained their stomatal conductance and showed lower foliar damage than FM in monoculture. Since FM was not able to access deep-soil moisture in our experimental set up, we assign the improved water relations of FM to bioirrigation by PP. These results are in line with previous studies showing that plants growing in close vicinity of a HL performing plant can benefit from the process of HL with respect to their water relations [41–44]. Sekiya et al. [44] performed a split-root experiment to demonstrate that neighboring shallow-rooted plants had access to deep soil moisture lifted by deep-rooted donor plants and as a result had higher stomatal conductance. Similar findings were reported by Dawson [2] showing that shallow-rooted plants growing next to Maple trees conducting HL maintained high stomatal conductance and were able to utilize hydraulically lifted water.

Contrary to our expectation, the presence of a CMN between PP and FM and PGPRs did not facilitate higher stomatal conductance of FM during the drought period through the promotion of bioirrigation. This is in contrast to a previous study [20] that involved the same species but an experimental design with smaller pots, where FM in treatments with CMN had significantly higher stomatal conductance than FM without CMN during drought. Also Saharan et al. [18] has reported that the presence of a CMN in a PP–FM intercropping system alleviates the negative effect of drought on FM. It is possible, that the missing effect of a CMN on stomatal conductance of FM during drought could be because the mycorrhizal hyphae did not effectively connect the rhizosphere of two plants since PP and FM plants were placed 15 cm apart. The effectiveness by which a CMN facilitates bioirrigation could thus depend on the spatial arrangement of the rooting systems of intercropped plants and on the distance a CMN has to bridge between two rhizospheres. However, we did observe that the presence of a CMN in combination with PGPRs significantly reduced the total foliar damage of FM during drought suggesting that the facilitation of water supply to FM by PP were yet positively affected by the CMN.

### Balancing competition and facilitation

The facilitative effect of bioirrigation during the drought period did not translate into an increased FM biomass at the end of the experiment, nor was there a positive effect of a CMN on FM biomass. Rather, plant biomass (shoot and root) of FM was significantly reduced in intercropping treatments, which was even enhanced in the presence of a CMN. This is most likely due to interspecific competition for soil resources (water and nutrients) with PP (Fig 7A & 7B). In fact, we detected strong competition between FM and PP for soil moisture in the topsoil layer before or just after the onset of drought. Soil moisture in intercropping treatments became for example more rapidly depleted than in FM monoculture with the onset of drought and dropped to the low levels of the monoculture treatments five weeks earlier. The apparent competition for water can also explain why we did not observe any beneficial effects of intercropping on the growth of FM during drought (Fig 5). Plants in the FM monoculture treatments grew well at the beginning of the drought period, most likely due to an absence of interspecific competition for water in the topsoil layer and a relatively high soil moisture despite the drought treatment (Fig 5C). FM in the intercropping treatments (both with and without CMN) did not continue to grow in height after the onset of drought. The competitive effects on FM were enhanced by the presence of a CMN and PGPRs that primarily promoted the growth of PP, in particular below-ground.

Similar results of facilitation and competition between deep-rooted and shallow-rooted plants have been reported, mostly in agroforestry systems [15,45,46]. Prieto et al. [43] reported that *Retama sphaerocarpa* L. conducts HL and supports establishment (survival) of seedlings of shrub *Marrubium vulgare* under its canopy but biomass of seedlings decreased significantly due to competition for soil resources, showing that competitive effects between intercropping partners were stronger than facilitative effect of HL.

### Effects of intercropping and CMN on yield

Despite the reported competition between FM and PP, our study yet shows positive intercropping effects between FM and PP. These effects were, however, only significant in the absence of a CMN and PGPRs. The partial LER values of PP in treatments without CMN had LER values of 0.89 and 0.83, while LER values of FM were close to 0.50 (Table 8). This suggests that PP benefits from intercropping irrespective of presence or absence of CMN and PGPRs, while FM does not benefit from intercropping but faces neutral effects or even suppression in the presence of PP when a CMN is present. Although we have indications that the presence of a CMN promotes the facilitative effects of PP on FM during drought, the presence of a CMN yet reduced the LERs, in particular of FM (Table 8). The suppression of FM yield is likely the result of enhanced competition for water where CMN and PGPRs facilitated growth of its host (PP). LER values of this study ranged from 1.17 to 1.37 which fits into often reported range for additive intercropping involving tropical and subtropical crops [47–50]. As such, our study highlights the effectiveness of intercropping for survival, but at the same time it shows that competition and facilitative effects have to be carefully balanced if CMN-facilitated intercropping is to be converted in higher yields on the field.

## Conclusions

Our results demonstrate that PP can support the water relations and survival of FM during a drought period. Our results are thus proof of the “bioirrigation” concept. The establishment of a CMN through inoculation with AMF did, however, not affect the water relations of FM during drought as strongly as we had expected from previous work. Yet, the presence of a CMN in intercropping treatments did reduce drought-induced foliar damage of FM suggesting that facilitative effects between PP and FM were enhanced by a CMN. In contrast to the facilitative effects under drought, PP exerted strong competitive effects on FM before the onset of drought under well-watered conditions, most likely due to competition for water in the topsoil layer. This hindered growth and biomass production of FM when intercropped with PP, an effect that was even enhanced by the presence of a CMN. In summary, the results from our study indicate that in intercropping deep-rooted PP may potentially act as a "bioirrigator" for shallow-rooted crops such as FM and that this effect is modulated by the presence of a CMN. However, the interspecific competition between PP and FM can be severe under ambient water supply and has to be considered in order to avoid yield loss. This is in particular in the presence of a CMN, as the CMN seems to enhance the competitive ability of PP. In more general terms, our study shows that the extent by which the antagonistic effects of facilitation and competition are expressed in an intercropping system strongly depends on the availability of resources, which in the case of the present study was water and the presence of a CMN. Our study shows that competitive and facilitative processes should be assessed in the design of intercropping systems in order to promote crop yields in such systems.

## Supporting information

S1 Table Raw means and standard error values of Fig 4. (XLSX)

S2 Table Raw means and standard error values of Fig 5. (XLSX)

S3 Table Raw means and standard error values of Fig 6. (XLSX)

S4 Table Raw means and standard error values of Fig 7. (XLSX)
